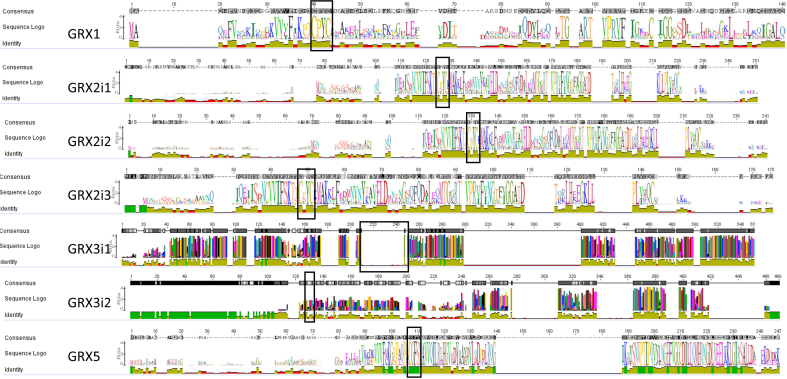# Glutaredoxin: Discovery, redox defense and much more

**DOI:** 10.1016/j.redox.2021.101975

**Published:** 2021-04-20

**Authors:** Fernando T. Ogata, Vasco Branco, Filipa F. Vale, Lucia Coppo

**Affiliations:** a Department of Biochemistry/Molecular Biology, CTCMol, Universidade Federal de São Paulo, Rua Mirassol, 207. 04044-010, São Paulo - SP, Brazil; b Research Institute for Medicines (iMed.ULisboa), Faculty of Pharmacy, Universidade de Lisboa, Av. Prof. Gama Pinto, 1649-003, Lisboa, Portugal; c Host-Pathogen Interactions Unit, Research Institute for Medicines (iMed-ULisboa), Faculty of Pharmacy, Universidade de Lisboa, Lisboa, Portugal; d Division of Biochemistry, Department of Medical Biochemistry and Biophysics, Karolinska Institute, Solnavägen 9, SE-17165, Stockholm, Sweden

**Keywords:** Glutaredoxin, Redox regulation, Glutathionylation, Deglutathionylation, Iron homeostasis, Grxs phylogenetics

## Abstract

Glutaredoxin, Grx, is a small protein containing an active site cysteine pair and was discovered in 1976 by Arne Holmgren. The Grx system, comprised of Grx, glutathione, glutathione reductase, and NADPH, was first described as an electron donor for Ribonucleotide Reductase but, from the first discovery in *E.coli*, the Grx family has impressively grown, particularly in the last two decades. Several isoforms have been described in different organisms (from bacteria to humans) and with different functions.

The unique characteristic of Grxs is their ability to catalyse glutathione-dependent redox regulation via glutathionylation, the conjugation of glutathione to a substrate, and its reverse reaction, deglutathionylation. Grxs have also recently been enrolled in iron sulphur cluster formation. These functions have been implied in various physiological and pathological conditions, from immune defense to neurodegeneration and cancer development thus making Grx a possible drug target.

This review aims to give an overview on Grxs, starting by a phylogenetic analysis of vertebrate Grxs, followed by an analysis of the mechanisms of action, the specific characteristics of the different human isoforms and a discussion on aspects related to human physiology and diseases.

## 1 Introduction

Glutaredoxin, Grx, was discovered by Arne Holmgren in 1976 in a mutant *Escherichia coli, E.coli,* strain lacking thioredoxin, Trx [1]. At that time, Trx was the only known natural reducing system for Ribonucleotide Reductase, RNR [2]. Structurally, Grxs are small thiol proteins of the thioredoxin-fold superfamily containing an active site with the sequence motif CXXC/S. Although Trx and Grx function as hydrogen donors for RNR and have the same motif in their active site, many of their functions do not overlap. The sphere of Grx functions continues to grow, and almost 50 years later it is clear that Grx is much more than a back-up system for Trx as first observed [3]. In the following sections we will cover some of the most important aspects of the Grxs.

## 2 Diversity of Glutaredoxin in vertebrates

A number of interesting studies have been published concerning the diversity of Grxs in different domains of life [[4], [5], [6], [7]], a diversity that reflects the multitude of functions versing from regulation of development [[8], [9], [10], [11]], metabolism [12], stress response [13] and environment adaptation [14,15]. Hereafter, we provide a more detailed analysis on the phylogenetic relations between human Grx isoforms and other vertebrate Grxs.

Genes coding for proteins with similar functions are likely to co-evolve and have homologs in the same subset of organisms [4]. To preserve function and structure, protein sequences tend to be conserved over much longer periods than the individual codons. The maximum-likelihood phylogenetic analysis of the Grxs present in vertebrates (Taxonomy id 7742), including Grx1, three isoforms of Grx2, two isoforms of Grx3 and Grx5 (Fig. 1, Fig. 2, Fig. 3, Fig. 4) show *E. coli* sequences as an outgroup, i.e. the topology of the tree places *E. coli* Grxs in a more distantly related (grey) cluster which supports a clear segregation between eukaryote and prokaryote Grxs. Even within eukaryotes there is substantial variation. In fact *D. melanogaster* Grxs tend to follow the same pattern, clearly segregating from the vertebrate Grxs (Fig. 1, Fig. 2, Fig. 3, Fig. 4, Fig. 1, Fig. 2, Fig. 3, Fig. 4 and Fig. 1, Fig. 2, Fig. 3, Fig. 4), except for all isoforms of Grx2. Surprisingly, for the three Grx2 isoforms (Fig. 2) the tree topology (indicating the relatedness patterns among taxa) showed that *D. melanogaster* is closer to amphibian and bony fish than birds, although, the long branch length of *D. melanogaster* sequences suggests a longer evolutionary time. In general, the phylogeny is consistent with the expected segregation, i.e., segregating vertebrates from D*. melanogaster*. The tree inconsistency in the case of Grx2 may be due to a distinct evolution of the gene coding for Grx2 in relation to the genome, but we may not disregard the difficulty of ascertaining the divergence time of organisms that diverged long ago, like insects and vertebrates.

**Fig. 1:**
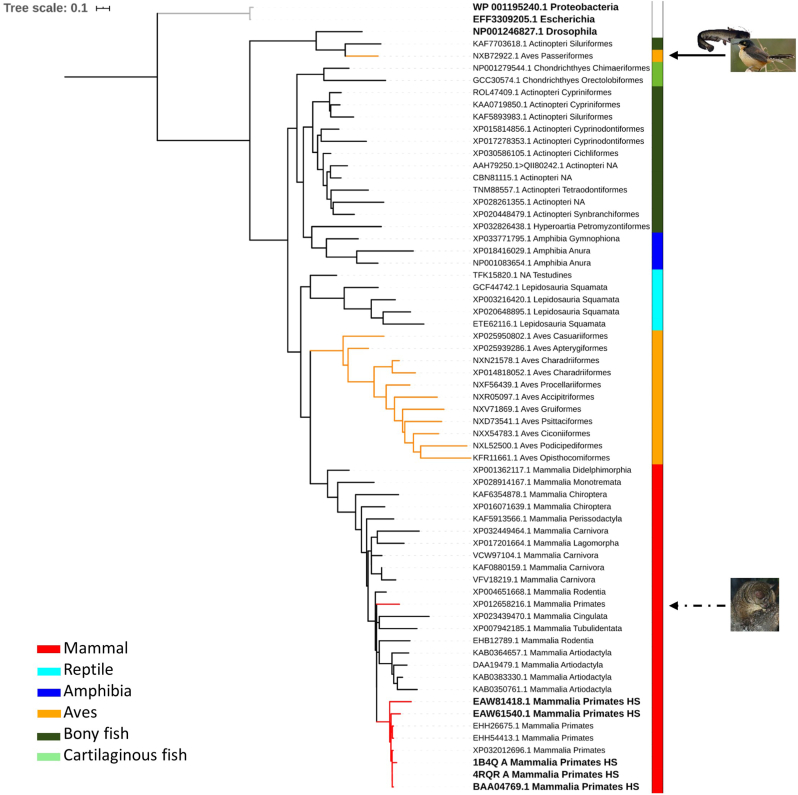
Maximum-likelihood phylogenetic tree of Grx1 protein sequences of representative species of vertebrates classes. Bold taxa indicate human or outgroup glutaredoxins. Red tree nodes indicate primates, and yellow nodes indicate bird’s glutaredoxins. Grey nodes indicate the *E. coli* outgroup. Vertebrate classes are colored coded by the lateral strip. Continuous black arrows indicate heterogeneous bird clades, and discontinuous back arrows indicate heterogeneous primate’s clades, with images of the species in question. A collection of sequences was retrieved from Genbank after performing a BLASTP [228] of Grx1 (NP_001112362.1 glutaredoxin-1 [Homo sapiens]). The number of sequences retrieved after BLASTP search varied between 500 and 1000, using as criterion having at least one species belonging to Mammal, Bird (Aves), Bony/Cartilaginous fish, Reptile and Amphibia. Each taxon in the phylogenetic tree is displayed by the accession number and the taxonomic class and order. Human sequences are identified with HS. Additionally, since Grxs are ubiquitous enzymes [17], we included *E. coli* and *Drosophila melanogaster* Grxs as outgroups, choosing the two most similar sequences found by BLASTP for each of the Grxs. Each protein sequence was aligned using MAFFT version 7 [229]. Identical sequences were removed before constructing maximum-likelihood phylogenetic trees with FastTreeMP [230]. The Interactive Tree of Life (iTOL) v4 [231] was used to visualize and annotate trees. To reduce the tree complexity, the leaves that contribute the least to the tree diversity were pruned using Treemmer v0.2 [232]. (For interpretation of the references to color in this figure legend, the reader is referred to the Web version of this article.)

**Fig. 2:**
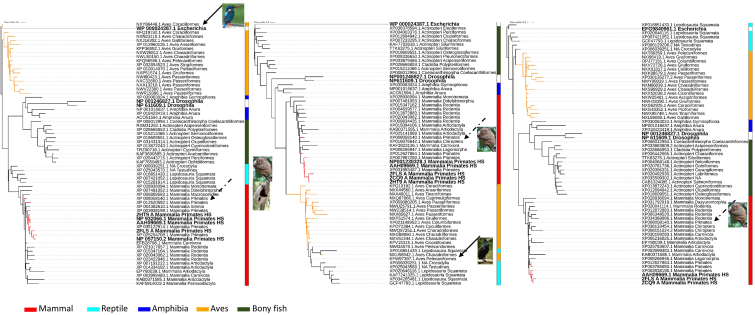
Maximum-likelihood phylogenetic tree of Grx2 protein sequences of representative species of vertebrates classes. Bold taxa indicate human or outgroup glutaredoxins. Isoforms 1, 2 and 3 from the left to the right. Red tree nodes indicate primates, and yellow nodes indicate bird’s glutaredoxins. Grey nodes indicate the *E. coli* outgroup. Vertebrate classes are colored coded by the lateral strip. Continuous black arrows indicate heterogeneous bird clades, and discontinuous back arrows indicate heterogeneous primate’s clades, with images of the species in question. A collection of sequences was retrieved from Genbank after performing a BLASTP [228] of Grx2 isoform 1 (NP_057150.2 glutaredoxin 2 isoform 1 [Homo sapiens]; Grx2b), Grx2 isoform 2 (NP_932066.1 glutaredoxin 2 isoform 2 precursor [Homo sapiens]; Grx2a), Grx2 isoform 3 (NP_001230328.1 glutaredoxin 2 isoform 3 [Homo sapiens]; Grx2c) and the phylogenetic tree constructed in a similar manner as for Fig. 2. (For interpretation of the references to color in this figure legend, the reader is referred to the Web version of this article.)

**Fig. 3:**
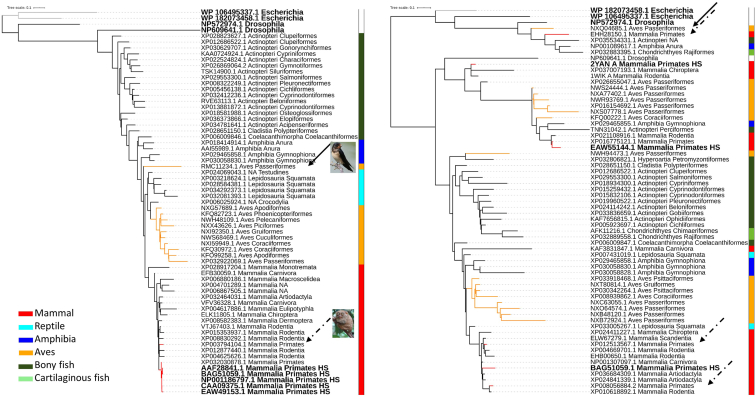
Maximum-likelihood phylogenetic tree of Grx3 protein sequences of representative species of vertebrates classes. Bold taxa indicate human or outgroup glutaredoxins. Isoforms 1 and 2 from the left to the right. Red tree nodes indicate primates, and yellow nodes indicate bird’s glutaredoxins. Grey nodes indicate the *E. coli* outgroup. Vertebrate classes are colored coded by the lateral strip. Continuous black arrows indicate heterogeneous bird clades, and discontinuous back arrows indicate heterogeneous primate’s clades, with images of the species in question. A collection of sequences was retrieved from Genbank after performing a BLASTP [228] of Grx3 isoform 1 (NP_006532.2 glutaredoxin-3 isoform 1 [Homo sapiens]), Grx3 isoform 2 (NP_001308909.1 glutaredoxin-3 isoform 2 [Homo sapiens]) and the phylogenetic tree constructed in a similar manner as for Fig. 2. (For interpretation of the references to color in this figure legend, the reader is referred to the Web version of this article.)

**Fig. 4:**
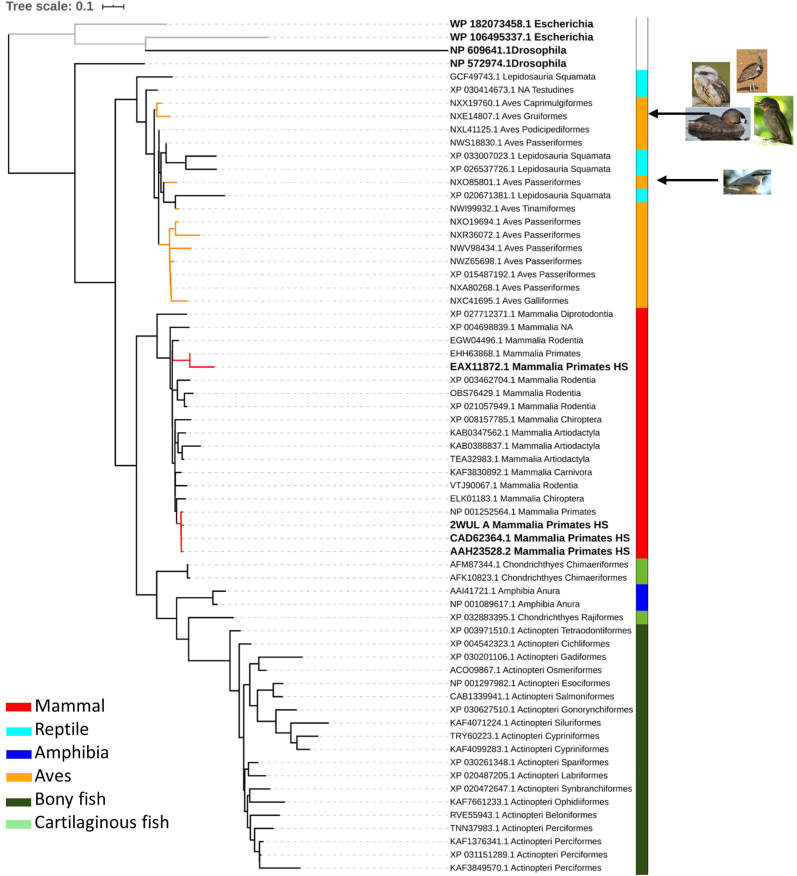
Maximum-likelihood phylogenetic tree of Grx5 protein sequences of representative species of vertebrates classes. Bold taxa indicate human or outgroup glutaredoxins. Red tree nodes indicate primates, and yellow nodes indicate bird’s glutaredoxins. Grey nodes indicate the *E. coli* outgroup. Vertebrate classes are colored coded by the lateral strip. Continuous black arrows indicate heterogeneous bird clades, with images of the species in question A collection of sequences was retrieved from Genbank after performing a BLASTP [228] of Grx5 (NP_057501.2 glutaredoxin-related protein 5, mitochondrial precursor [Homo sapiens]) and the phylogenetic tree constructed in a similar manner as for Fig. 2. (For interpretation of the references to color in this figure legend, the reader is referred to the Web version of this article.)

Considering vertebrate Grxs tree topology, these are largely consistent with the phylogenetic relationships of the taxonomic classes of vertebrates, i.e., the trees clearly segregate clades by taxonomic classes (Fig. 1, Fig. 2, Fig. 3, Fig. 4). However, notable exceptions are found for Aves class (birds) for Grx1, Grx2 and Grx3, with several orders (Fig. 1, 2 and 3), forming distinct clades, thus not supporting a unique monophyletic birds’ group (for details refer to Fig. S1, S2 and S3) as previously described [16].

**Fig. 5:**
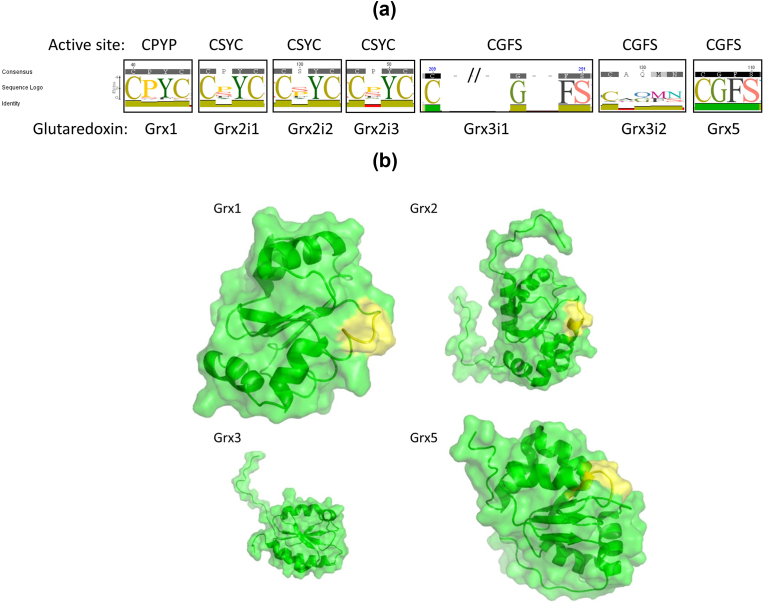
Sequence Logos of the active sites of glutaredoxins present in vertebrates obtain from a multiple sequence alignment of 500 to 1000 glutaredoxins sequences. Active site described for each glutaredoxin. Consensus: most common amino acid found in the alignment. Sequence Logo representing the conservation at each position. Identity: Green: 100% identity, Greenish-brown: at least 30% and under 100% identity, Red: below 30% identity. Sequences logos were produced from multiple sequence alignments, where the height of the amino acid symbol represents its relative frequency and the height of the stack represents the sequence conservation at each position [233]. **B)** Representation of the tridimensional surface and secondary structure of human glutaredoxins evidencing the active site in yellow (Protein Data Base structures 1B4Q, 2CQ9, 2DIY and 2MMZ for GRX1, GRX2, GRX3 and GRX5, respectively) visualized using PyMOL 2.4. (For interpretation of the references to color in this figure legend, the reader is referred to the Web version of this article.)

Finally, Grx5 is highly conserved in eukaryotes and prokaryotes. Accordingly, the phylogenetic tree for this isoform displayed a topology segregating by vertebrate classes (Fig. 5) with *E. coli* and *D. melanogaster* Grx5 like proteins forming clear outgroups. The *GLRX5* gene may have originated in bacteria resulting from the translocation of the endosymbiotic mitochondrial genome into the nuclear genome. Indeed the Grx5 protein has a mitochondrial signal sequence to target for the organelle where it operates [17]. Additionally, two clear clades are observed (Fig. 4 and S4), one including birds and reptiles and the other the remaining vertebrates. Taken together the phylogenetic analysis showed that Grxs were acquired long before the speciation event, i.e., the species divergence from a common ancestor, but in the case of birds the Grx origin may be variable.

Interestingly, not all Grxs from primates cluster together. Remarkably, the Grx1 like protein of the primitive *Otolemur garnettii* (nocturnal and arboreal primate endemic in Africa) clusters apart from the others, pointing to a much earlier divergence from other primates [18,19], evidenced by the higher sequence similarity of rodent’s Grx1 (Fig. 1). Similarly, Grx2 isoforms also segregate primates, namely *Carlito syrichta* (Philippine tarsier) and *Propithecus coquereli* (sifakas) in a distant clade; and Grx3 isoform 1 of *O. garnettii* is also segregated in a different clade. It appears that the proteins Grx1, Grx2 and Grx3 can discriminate early divergence among primates, working like a “molecular clock”. The small branch lengths among mammal Grx5 clade (Fig. 4) represents a short evolutionary time (sequence conservation).

Grxs are characterized by highly conserved active sites (Fig. 5A and B and S5), particularly the active site of Grx5 reaches 100% conservation, representing a strict conserved group with clear homologs in all analysed vertebrate taxa. Only the active site of isoform 2 of Grx3 showed poorer conservation, which agrees with the phylogenetic tree obtain for this isoform, where the clustering did not sharply depict vertebrates’ taxonomic classes.

## 3 General aspects on glutaredoxins

### 3.1 Functions and regulations

Grx functions comprise thiol-redox properties, dehydroascorbate reductase and transhydrogenase activity, de-nitrosylation and in part cystine conversion. However, the thioltransferase activity and iron homeostasis appear to be the most critical functions for physiology *in vivo* [3,20]. It has to be underlined that biochemically, the thioltransferase activity is different from the GSH transferase (GST activity) because the reactions catalysed by Grx are oxidoreduction rather than transferase reactions [21].

Protein thiolation by GSH (glutathionylation; PSSG) – i.e. the formation of a mixed disulphide between GSH and a protein Cys - was initially described as a non-specific modification induced by strong oxidative conditions and later considered a protective mechanism that avoids over and irreversible oxidation of protein Cys residues during an oxidative stress event. This is a dynamic process where Grxs play a major regulatory function.

The concept of glutathionylation as major mechanism of oxidative signal transduction, first postulated by Mieyal et al. in 1995 [22], is supported by the increasing number of proteins known to be functionally modulated by glutathionylation, via Grx. Proteins so far identified that are susceptible to glutathionylation can be categorized into six distinct clusters: cytoskeletal, glycolysis/energy metabolism, kinases and signalling pathways, calcium homeostasis, antioxidant enzymes, and protein folding [23]. The criteria for glutathionylation as a regulatory mechanism are (i) glutathionylation occurs at a specific cysteine and (ii) glutathionylation is reversible, thus allowing reversion of the signal [24]. Grxs (in particular the isoforms Grx1 and Grx2) catalyse the deglutathionylation reaction much more effectively than other thioltransferases such as Trx or protein disulphide isomerase [25] allowing the reversibility of the signal. Specific examples of the role of Grx in the regulation of the glutathionylation are given in subsequent sections.

Despite research efforts to understand the mechanism by which the redox signal is transmitted from oxidant to specific proteins and in particular how Grx is activated to reverse or put forward glutathionylation, much remains to be elucidated.

The monothiol Grxs, Grx3 and Grx5, are evolutionarily highly conserved from bacteria to mammals and are important in iron trafficking, iron homeostasis and storage [[26], [27], [28]]. Grx3 and Grx5 form an iron-sulphur complex and recent studies indicated that both isoforms can transfer the iron to specific proteins and to the members of the iron assembly machinery (see section 2.3.3 and 2.3.4 for more details). Unlike the dithiol forms, the participation and role of the monothiol Grxs in redox chemistry remains controversial due to several observations. First, monothiol Grxs (active site sequence CXXS) cannot deglutathionylate target proteins. Furthermore, yeast Grx5 does not display redoxin activity using the classic Grx substrate HED (Hydroxyethyl disulphide [29]), does not have activity as dehydroascorbate reductase or GSH peroxidase activities [30].

### 3.2 Catalytical mechanisms

**Fig. 6:**
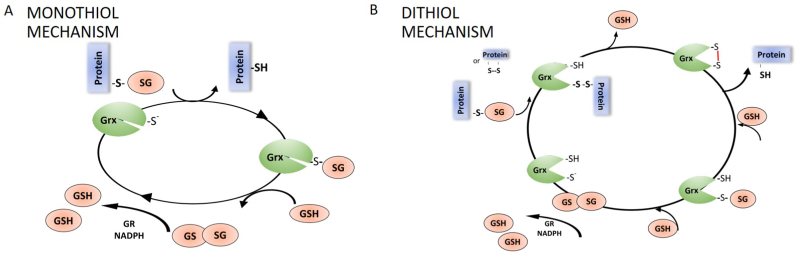
**Schematic representation of Grx catalytic mechanisms.** (A) Monothiol mechanism of Grx. This mechanism depends only on the N-terminal active site cysteinyl residue that attacks the GSH moiety (1) and forms a GSH-mixed disulfide intermediate (2). Thus, the substrate is reduced. A molecule of GSH is required to reduce the Grx-S-SG mixed disulfide (3) Electrons are transferred from NADPH via GR to reduce GSSG (4) (B) Dithiol mechanism of Grx. This mechanism depends on both active site cysteines. The N-terminal active site cysteine has a low pKa value, allowing the initiation of a nucleophilic attack preferentially on mixed disulfides with GSH with formation of a covalently bound mixed disulfide intermediate (1). In the second step the C-terminal active site cysteine reduces the mixed disulfide releasing the reduced protein (2). The oxidized Grx is reduced by two molecule of GSH (3 and 4) electrons are transferred from NADPH via GR to reduce GSSG (5) Grx: glutaredoxin; GSH: reduced glutathione; GSSG: oxidized glutathione; GR: glutathione reductase; NADPH: nicotinamide adenine dinucleotide phosphate.

Glutaredoxins can be classified based on different parameters such as sequence similarity, domain architecture, quaternary structure, enzymatic activity, and iron–sulphur cluster binding. Firstly, considering the number of cysteine residues in the active site, Grxs are divided into two groups: monothiol (CXXS) and dithiol (CXXC) (Fig. 6). Reduction of glutathionylated proteins (PSSG) by monothiol mechanism [[31], [32], [33]] (active site CXXS, Fig. 6A), initiates with a nucleophilic attack of the N-terminal cysteine which is characterized by high accessibility and has a low pKa (5.0 in yGrx5, 3.9 in hGrx1 and 4.8 in hGrx2) [3,30,[34], [35], [36]]. The GSH moiety is transferred between the substrate protein and Grx, releasing a glutathionylated-Grx and a reduced substrate protein (Fig. 6A–1). The Grx-SG intermediate is cleaved by a GSH molecule (Fig. 6A–2), forming reduced Grx (Fig. 6A–3) and one molecule of GSSG that is reduced by GR using NADPH as an electron donor to provide the reducing power to continue running the cycle (Fig. 6A–4) [37,38].

In the dithiol mechanism (active site CXXC) [34,39,40] (Fig. 6B), the Grx N-terminal cysteine attacks the mixed disulphide, PSSG, but releases GSH as the leaving group (Fig. 6B–1). The Grx-protein intermediate is reduced by the second C-terminal active-site cysteine of Grx, leading to oxidized Grx and a reduced protein target (Fig. 6B–2). GSH reduces the oxidized Grx following the mechanism showed in Fig. 6B–3 and 6B-4. Both Grx mechanisms are critically dependent on the availability of reduced GSH to drive deglutathionylation reaction.

The C-terminal sequences are slightly different between dithiol and monothiol Grxs [32] and it seems to affect GSH binding and may explain the biochemical differences between the two mechanisms of action. Interestingly, all dithiol Grxs tested may also employ the monothiol mechanisms, but how mechanism selection occurs and under which physiological conditions it happens, is not known.

### 3.3 Human Glutaredoxin family

The two most studied human Grxs are the dithiol isoforms Grx1, which mainly exists in the cytosol, and Grx2, which localizes to mitochondria, cytosol or nucleus depending on gene splicing [41,42] (Table 1). Unlike Grxs found in prokaryotes which contain only the classic mono-cysteine active site, eukaryotic monothiol Grxs are more diverse and may have either the classic mono-cysteine domain or the so-called “hybrid Trx-Grx-domain” [4]. Humans utilize both mono-cysteine domains (Table 1): Grx5 with the classic mono-cysteine domain [43] localized in the mitochondria; and Grx3, also called PICOT (for Protein kinase C theta, PKC-interacting cousin of Trx) [44,45] with a Trx-Grx structure, localized in the cytosol.

#### 3.3.1 Glutaredoxin1-Grx1

The gene for human Grx1 (*GLRX*) consists of three exons and three introns, localized to chromosome 5q15. Human Grx1 with a Cys-Pro-Tyr-Cys (CPTC) active site is mainly a cytosolic protein but it has been reported in low concentration also in nucleus [46,47] and mitochondrial intermembrane space [48]. Grx1, unlike Trx, is not an essential protein since transgenic or knockout mice are viable and with a life span similar to wild type mice (for a review on Grx mice models see Ref. [27]).

In addition to the active site cysteines, human Grx1 contains three additional cysteine residues that can be oxidized. These extra-cysteine functions are still unknown, but they are thought to affect enzymatic activity [49].

A recent study measured the redox potential of human and *E.coli* Grx [40] and showed that both enzymes had a reduction potential in between a classic disulphide reductase proteins (Trx) and a model of protein dithiol oxidase (disulphide bond formation protein A, DsbAs). Therefore, the activity of Grx1 seems to depend on the redox state of cells, particularly on the GSH/GSSG ratio. The reduction potentials for both human and *E.coli* Grx1 were demonstrated to vary between internal disulphide and glutathionylated form depending on the GSH concentrations [50]. Even if the ratio GSH/GSSG seems to be an important factor in the Grx1 activation, the detailed mechanism and how Grx1 can be activated and how recognized target proteins is not understood.

In general, loss of Grx1 leads to redox perturbation of cytosolic proteins and increase the concentration of glutathionylated proteins, which disrupt normal redox signalling. Example of proteins that change function upon glutathionylation are listed in Table 2. In addition to deglutathionylation activity, Grx1 has also been shown to be able to de-nitrosylate protein Cys and prevent the proapoptotic effect of nitric oxide in tumour cell lines [51,52] and in cardiomyocyte [52].

**Table 1:** Human Grx localization.

Human Grx	Active site motif	Mass (kDa)	Localization	Gene
Grx1	CPYC	12^#^	Cytosol*, mitochondrial intermembrane space, nucleus	**GLRX**
Grx2	CSYC	18	Mitochondria* (Grx2a), cytosol (Grx2c), nucleus (Grx2b)	**GLRX2**
Grx3 (PICOT)	CGFS	37	Cytosol*, nucleus	**GLRX3**
Grx5	CGFS	17	Mitochondria*, cytosol	**GLRX5**

*cell compartment in wich the isoform was first described. ^#^small differences between the different splicing variants.

**Table 2:** Example of proteins function affected by glutathionylation and recognized or possible target of Grx regulation.

Proteins regulated by glutathionylation	Glutathionylation Induced Functional Changes
GAPDH^#^ [[53], [54], [55]]	Inhibition
NF-kB^#^ [[56], [57]] (p65 [[58], [59]]; P50 [60])	Inhibition
PTP1B^#^ [61]	Inhibition
c-Jun [[62], [63]]	Inhibition
Rac-1 [[64], [65]]	Inhibition
Creatine kinase^#^ [[66], [67]]	Inhibition
Actin^#^ [[68], [69], [70], [71]]	Activation
Akt^#^ [[72], [73]]	inhibition
Fas (CD95; Apo-1) ^#^[74]	Activation
AMPK^#^ [[75], [76]]	Activation
Estrogen receptor alpha [77]	Inhibition
Catalase [78]	inhibition
Ryanodine receptor 2^#^ [[79], [80]]	Activation
Complex I ^#^ [81]	inhibition
Sirtuin-1^#^ [[82], [83]]	Inhibition
Mitofusin [84]	Activation
IKK-Beta^#^ [85]	Inhibition

# Grx is reported to regulate the glutathionylation.

Two studies, in particular, stand out as important reports on the effects of glutathionylation and Grx1 regulatory activity.

The study by Shao and co-authors, using Grx1^−/−^ mice model, showed the importance of this redoxin in regulating hepatic lipid homeostasis and preventing fatty liver disease via regulation of glutathionylation of the histone deacetylase sirtuin1. In fact glutathionylated sirtuin1 has a decreased activity that leads to hyperacetylation and activation of sterol regulatory element-binding protein, SREBP-1, leading to upregulation of enzymes involved in lipid synthesis [82]. The mice displayed a non-alcoholic fatty liver disease indicating a potential role for Grx in the progression of fatty liver diseases.

The second study by Anathy and co-authors [86] showed decreased Grx1 activity in lung tissues from human subjects with idiopathic pulmonary fibrosis, which correlates with an increase in PSSG and disease severity. A more detailed mechanistic analysis using a transgenic Grx^-/-^ mouse model showed that the induction of lung fibrosis generated substantially higher PSSG that could be reversed by exogenous administration of recombinant Grx1, thus opening a new therapeutic approach for lung fibrosis.

#### 3.3.2 Glutaredoxin 2-Grx2

The gene for human Grx2 (*GLRX2*) consists of five exons and four introns localized to chromosome 1q31.2–31.3 [87,88]. Human Grx2 is only 34% identical to Grx1 [42] and lacks one of the extra cysteines present in Grx1, Cys-83 [42]. Moreover, Grx2 is about 20 times less abundant than Grx1 [89]. The Grx2 sequence contains three characteristic regions of the Grx family: the dithiol/disulphide active site, CSYC, encoded by exon III; the GSH binding site, encoded by exons III and IV and a hydrophobic surface area. Two alternatively spliced Grx2 mRNA isoforms were identified; exon Ia encodes a mitochondrial translocation signal while exon Ib has been suggested to be targeted to the nucleus [42,87,90]. Jurado et al. [91] detected the expression patterns of genes coding for Grx2 in different organs and stage of life in mice. The mitochondrial isoform (Grx2a) accounted for 40% of Grx2 mRNAs in all organs examined except the testis (only 1% of Grx2a) with an even higher level during embryonic development (53% at embryonic day (ED)11), indicating the critical role of Grx2 in mitochondrial redox regulation at this developmental stage.

In zebrafish, Grx2 is an essential protein for successful embryogenesis, regulating neuronal survival and axon growth [92] as well as angiogenesis through S-glutathionylation of the histone-deacetylase sirtuin1 [93,94]. Unlike zebrafish, mice lacking Grx2 are viable, however, they develop heart hypertrophy and fibrosis and become hypertensive [95]. In humans, low levels of Grx2 transcripts are associated with fibrosis, hypertrophy and infarctions of the left ventricle [96], thus stressing the key role of Grx2 in vertebrate heart physiology.

Like Grx1, Grx2 catalyses the reduction of GSH-mixed disulphides with higher affinity but with lower turnover rates [97]. However, the two proteins behave differently in response to the oxidative environment. While Grx1 is inhibited when the additional structural cysteine residues become oxidatively modified [49,98], Grx2 is activated. The different response to oxidative conditions is due to the ability of Grx2 to form Fe–S clusters. Grx2 is thus very resistant to oxidative inactivation compared to other thiol-redox proteins [49]. Lillig and co-authors characterized the Grx2 Fe–S complex and described that the two cysteines outside the active site, Cys-28 and Cys-113, coordinate the cluster. The [2Fe–2S]-bridged dimer is enzymatically inactive but oxidative stress, increased GSSG concentrations or reduced availability of GSH for the [Fe–S] complex coordination, results in degradation of the cluster and formation of enzymatically active Grx2 monomers. This may suggest that the [Fe–S] clusters act as sensors for Grx2 activity under oxidative conditions [98].

Another peculiar characteristic of Grx2 is that it can cycle and receive electrons from thioredoxin reductase1 (TrxR1) [97]**.** This demonstrate the existing crosstalk between the mammalian thioredoxin reductase- and glutathione reductase-driven pathways, which are classically considered independent reductase systems.

**Fig. 7:**
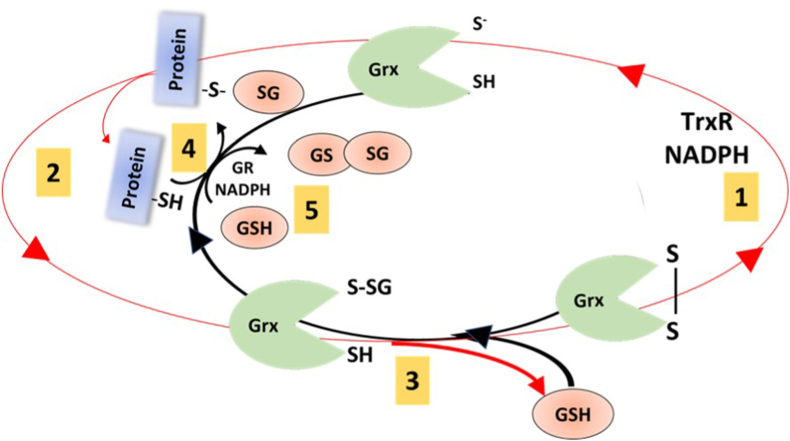
**Schematic representation of Grx2 activity**. This scheme shows how Grx2 catalyses the glutathionylation and deglutathionylation of protein thiols. The red circle represents alternative way of Grx2 reduction, via TrxR and NADPH (1) and PSSG reduction forming glutathionylated Grx2 (2). The second cysteine in the active site remove the GSH form the Grx2 leading to oxidized protein (3). However, glutathionylated Grx2 can transfer the GSH to a target protein (4 black arrow). Grx2 canonical dithiol mechanism is represented by step 2 (red arrow) and 5 (black arrow), mechanism explained in more details in Fig. 6B (Figure based on [28]). (For interpretation of the references to color in this figure legend, the reader is referred to the Web version of this article.)

In mitochondria, Grx2 has been shown to efficiently catalyse both glutathionylation and deglutathionylation of mitochondrial membrane protein cysteines such as Complex I [28,99,100] and SOD1 [101] (Fig. 7).

Complex I is particularly interesting since glutathionylation occurs only at the 75 and 51 kDa subunits. The 75-kDa subunit has 17 Cys residues, 6 of which are plausible candidates for glutathionylation. However, glutathionylation was only been detected on Cys-531 and Cys-704 indicating a high degree of specificity for this redox modification that inactivates Complex I. Glutathionylation and deglutathionylation of the Complex I subunit by Grx2 is highly dependent on the ratio of GSH/GSSG [28] indicating this redox modification may serve as a protective mechanism against oxidation of Complex I.

To better understand the role of Grx2 in physiological conditions, a trapping approach, utilizing Grx2 with the C-terminal resolving cysteine of Grx2 mutated, was performed by Schutte and co-authors in human HeLa cells and mouse tissues [102] which identified approximately 50 proteins as Grx2 glutathionylation targets. However, the regulation exerted by Grx2 has still to be investigated for the high majority of them.

#### 3.3.3 Glutaredoxin3-Grx3

Human Grx3 was initially described by Witte and co-authors [44]. The gene for human Grx3 (*GLRX3*) consists of 13 exons and is localized to chromosome 10q26.3 with pseudogenes located on the short arm of chromosomes 6 and 9. Grx3 has a unique domain structure consisting of an N-terminal Trx-homologous domain followed by two tandem repeats domains that resemble a Grx [32,44,45], despite the Trx homologous domains, no evidence has yet suggested that Grx3 can be reduced by TrxR.

Grx3 is an essential protein in mice. Indeed, homozygous Grx3^−/−^ mice die between ED12.5 and 14.5 [103]. At ED12.5 Grx3^−/−^ embryos show no obvious histological abnormalities but are smaller than wild type embryos and later develop haemorrhages in the head. Moreover, Grx3 is upregulated in wild type mice at ED9.5 just after the heart starts to beat and erythroid cells enter the circulation [104]. In rodents erythroid cells display a developmental progression and ED12.5 marks the terminal maturation and enucleation [105], probably representing the cause of the Grx3^−/−^ embryos' phenotype. Unlike the Trx^-/-^ and TrxR^-/-^ mice that are embryonic lethal but the conditional knockouts are well tolerated *in vivo* [20,106], the complete knock down of Grx3 leads also to cell death in cultures of human and mouse cell lines. These data suggest Grx3 has functions that cannot be compensated for by other proteins [107].

The first function discovered for Grx3 was related to Protein kinase C theta, PKC-θ, and in T-cells Grx3 colocalizes with PKC-θ, hence the name PICOT. Grx3 was discovered in stimulated Jurkat cells, where it attenuated the activation of mitogen-activated c-Jun N-terminal kinase and the transcription factors AP-1 and NF-κB [44]. Since, Grx3 is an iron-sulphur protein and is expressed in a wide variety of organs and tissues, some authors propose Grx3 as a redox sensor in signal transduction in response to reactive oxygen and nitrogen species [108]. The nuclear translocation of Grx3 after oxidative stress events supports this hypothesis [109]. However, a recent study has indicated that nuclear Grx3 contributes to the epigenetic regulation, silencing and remodelling of chromatin by regulating methylation of the myelin transcription factor 1 [110]. In a follow-up study Pandya & Isakov recently summarised the function of Grx3 in cell proliferation [107].

Cha and co-authors showed that Grx3 has a role in cardiomyocyte regulates contractility and cardiac hypertrophy and cardiomyocytes with heterozygous deletion of Grx3 (Grx3+/-) showed a decrease in Ca^2+^ sensitivity in cardiomyocyte filaments. The role of Grx3 in the ventricular function and cardiomyocyte contractility was demonstrated both by overexpression and depletion of Grx3 [103,111]. In Hela cells, Grx3 silencing decreased ferritin and iron regulatory protein I levels and upregulated transferrin receptors [108].

We are clearly only beginning to understand the functions of Grx3; therefore, we believe that a focus of future investigation on the interaction between Grx3 and different protein targets *in vitro* and *in vivo*, may lead to important discoveries about basic physiological mechanisms of life.

#### 3.3.4 Glutaredoxin 5-Grx5

The gene for human Grx5 (*GLRX5*) consists of 2 exons and is localized to chromosome 14q32.13 encoding for a mitochondrial protein, which is evolutionarily conserved [108] (see section 1). Grx5, a monothiol Grx, has a conserved active site sequence CGFS - of which Cys60 and Gly61 are essential - and shows high protein stability even upon amino acid substitution in key points of the protein [112] (Fig. 5A). Like other Grxs, Grx5 is involved in the biogenesis of iron-sulphur clusters.

Absence of Grx5 in yeast (yGrx5) was not lethal [113] but led to constitutive oxidative damage, cellular iron accumulation, and inactivation of iron–sulphur containing enzymes but not heme-containing enzymes [113]. A similar effect was reported in zebrafish and humans with addition impairment of heme biosynthesis and erythropoiesis [[114], [115], [116]]. Interestingly, two patients reported a silent mutation in the *GLRX5* gene, that results in non-syndromic microcytic congenital sideroblastic anaemia (cSA) [115,117] a condition that is associated with iron overload in mitochondria together with relative iron depletion in cytosol.

In the last few years, Grx5 has been studied as iron-sulphur cluster donor to the cytosolic iron sulphur cluster assembly machine (ISCA1) and to the mitochondrial iron-sulphur cluster assembly homolog (ISCA2). Banci and co-authors showed that human Grx5 can transfer [2Fe–2S] cluster to human ISCAs proteins. Moreover, it was described structurally how the [4Fe–4S] cluster is made upon interaction of human Grx5, ISCA1 and ISCA2 [[118], [119], [120]].

Grx5 has also been shown to form cluster with BolA-like protein family (DNA-binding regulator proteins) in the cytosol, for iron-sulphur protein maturation [121]. The complete function of Grx5 connected to the BolA-like proteins is far from being fully understood. This is due to several methodological problems as well as to the fact that Grx5 can form different heterocomplexes with the different BolA like proteins. In fact, the low stability of the BolA1–Grx5 cluster may have a role in electron transfer processes; the [2Fe–2S] BolA3–Grx5 heterocomplex could be more prone to [Fe–S] cluster trafficking because it is more kinetically labile [122]. Promisingly, some of the technical difficulties in measuring the heterocomplex, due to the inherent instability of the cluster, have begun to be addressed in the last year [123] opening new opportunities to better understand the role of Grx5 in iron trafficking. A study by Kim et al., in 2011 also questioned the role of Grx5 outside mitochondria and independent of iron metabolism, suggesting that hGrx5 could have a role, in reduction of PTEN protein [124].

## 4 Grxs in health and disease

In the last two decades the role of Grxs in physiology and pathology shifted from an antioxidant perspective to a modulatory one. For example, overexpression of Grx1 is known to protect cardiac functions after chronic ischemia, in contrast, Grx^-/-^ mice develop less cardiac hypertrophy compared to controls mice [125,126] (for a review on the role of Grx1 in cardiovascular diseases see Burns et al. [127]). Also, as mentioned in section 2.3.1, addressing Grx1 function seems to be a promising approach in lung fibrosis therapy. Moreover, in the context of Friedreich’s ataxia antioxidant proteins in particular Grx are studied to understand the redox homeostasis in the regulation of Nuclear factor erythroid 2-related factor 2, Nrf2, expression and in Fe–S cluster metabolism [128]. Among these different emerging involvements of Grx in pathological conditions we focus, here, on the role of glutaredoxins in central nervous system functioning and pathology and in cancer development.

### 4.1 Glutaredoxin function in the CNS

#### 4.1.1 Neuroprotection in neurons

Redox regulation in the Central Nervous System (CNS) is overly complex, but a fundamental aspect of CNS homeostasis regulation. Indeed, the metabolic demand for oxygen in the CNS accounts for 20% of the global demand of the body [129]. Besides energy production within cells, oxygen and reactive oxygen species (ROS) are fundamental for several developmental and cellular processes taking place in the CNS including control of cell proliferation and differentiation [130,131], signal transduction and inflammatory response [132]. In order to keep redox signalling under tight regulation, the CNS relies on the regulatory action of redox elements such as Grxs [133].

Grx1 is found throughout the brain of rodents and humans [134,135] and highly expressed in both neurons and glia cells. The high expression in glia cells suggests that Grx1 is deeply involved in supportive functions [136], such as, transcription factor’s activation in the nucleus [137,138] and protection of Complex I from oxidative damage at the mitochondria [139].

Likewise, Grx2 expression is ample in neurons and glia cells. An histochemical analysis of the rat brain [136], found Grx2 in the cytosol and nucleus of type I neurons, which could be an adaptation to cope with ischemia related damage. Also, the mitochondrial matrix localization of Grx2 can be inferred from *in vivo* experiments showing decreased Complex I activity when Grx2 is down-regulated [140].

Zebrafish embryos lacking Grx2 showed impaired neurodevelopment with widespread apoptosis of neurons and neural progenitors and lack of axonal outgrowth [92].

Besides this constitutive expression of Grxs, experimental evidence has demonstrated that the expression of both Grx1 and Grx2 can be up-regulated by various oxidative stress-causing stimuli, such as exposure to neurotoxicants like MPTP [140] emphasizing the importance of these enzymes in keeping redox homeostasis in the CNS.

Monothiol Grx3 and Grx5 are not as abundant in the CNS as their dithiol counterparts. Histochemical analysis of the mouse brain has shown that both enzymes are frequently found localized in the nucleus [137]. Indeed, Grx3 is actively translocated to the nucleus upon oxidative stress, where it acts to reduce ROS production and decrease the sensitivity to inflammatory stimuli [141]. A testimony to Grx3’s neuroprotective and anti-inflammatory role was the observation that, following thymoquinone treatment of LPS/IFN gamma-activated BV-2 microglia cells, Grx3 expression was more than 20-fold higher than in non-treated controls [142].

Since iron is essential for brain development, the role of Grx3 and Grx5 should be determinant for normal progression. However, experimental evidence establishing a direct link between their function, iron homeostasis and neurodevelopment in the brain is still lacking (see Berndt et al., 2018 for review [143]).

#### 4.1.2 Deglutathionylation in the CNS

The brain has considerably less available GSH than for example the liver. However, PSSG are abundantly formed in the CNS during oxidative events protecting sensitive cysteine residues [144]. The ability of Grxs enzymes to deglutathionylate proteins is essential to recover GSH from PSSG following oxidative insults [134]. Therefore, it is unsurprising to register such widespread and significant expression of Grx in brain cells.

A few examples of Grx involvement in the glutathionylation/deglutathionylation cycle in the CNS are hereafter described.

Upregulation of Grx was shown to restore Complex I activity, in an MPTP-induced Parkinson disease, PD, model. This is likely due to the deglutathionylation of key cysteine in the active site of Complex I [145]. In fact, down-regulation of mitochondrial Grx2 is linked to decreased Complex I activity [140], presumably due a to a reduction of deglutathionylation [28]. (See section 2.3.2).

Grxs seem to play an essential role in neuronal development by regulating the assembly of cytoskeletal filaments, since they are susceptible to overoxidation and post-translational modification [146]. Indeed, glutathionylation of Cys374 in actin prevents proper polymerization [71] and may lead to the accumulation of dysfunctional actin filaments [147]. On the other hand, complete depletion of ROS reduces the filamentous actin content. Thus, Grx activity is pivotal in maintaining the balance between glutathionylation and deglutathionylation, promoting neuronal survival and allowing extension of axonal processes [148]. Also, alpha and beta-tubulins, the microtubule constituents, have several cysteine residues that become glutathionylated under oxidative stress. The GSH/Grx system reverses this modification and allows for proper polymerization of tubulins [149]. Additionally, tubulin polymerization may be indirectly regulated via tau and Microtubule-associated protein 2 which also have cysteine residues susceptible to glutathionylation [149].

Phosphorylation of protein tyrosine residues is critical for signal transduction in the CNS [150]. Particularly, protein tyrosine phosphatase 1B (PTP1B) is susceptible to post-translational modification by several oxidative stimuli, which function as signal transducing mechanisms [61,151,152]. Specifically, glutathionylation occurs at active site Cys215 and is reversed by Grx, which thus regulates its activity and controls tyrosine residue phosphorylation.

Protein deglycase DJ-1, functions as a redox sensor with peroxiredoxin-like activity protecting neurons against oxidative stress [153,154]. Besides, in the nucleus DJ-1 sequesters the death-associated protein, Daxx, preventing its translocation to the cytosol to activate ASK-1 mediated cells death [155]. If Grx1 levels are down-regulated, DJ-1 function is hampered and Daxx mediated cell death takes place. This is attributed to glutathionylation of Cys53 and Cys106 in DJ-1 which target the protein for degradation [156]. Grx1 degluthationylase activity is thus essential to DJ-1 protective function.

In summary, current evidence points to glutathionylation as an important mechanism for maintaining redox equilibrium in the brain and regulating several proteins crucial for neurobiological processes. As a consequence, Grxs, being key modulators of protein-mixed disulphide formation assume a particularly relevant role in keeping redox homeostasis at CNS level.

#### 4.1.3 Aging and neurodegenerative diseases

Oxidative stress is regarded as one of the hallmarks of aging and neurodegeneration [157]. Likewise, reduced expression of antioxidant enzymes appears to be a common trait of the aging brain [158]. This means the aging brain presents not only a decreased ability to control oxidative stress but, as important, it has a decreased capability to regulate ROS mediated signalling, which is associated to the loss of cognitive function [158]. Similarly, to other antioxidant elements, the importance of Grx in aging appears to be no exception. As it was observed in Lou rats, a strain with longevity above average, Grx1 mRNA levels are essentially kept constant throughout lifespan. This correlates with a better cognitive performance than other strains where levels of Grx1 are known to decrease with age [159]. Higher Grx1 expression should aid in mitigating oxidative stress as seen in HT-22 murine hippocampal cells transfected with peptide-conjugated Grx1 where survival against a high concentration of H_2_O_2_ was enhanced, or as observed *in vivo* in a ischemia induced model [160]. Grx1 could thus be involved in the mitigation of the pro-oxidative effects associated to aging while at the same time securing the transduction of H_2_O_2_ mediated signalling.

In neurodegenerative diseases, increased expression of Grx has been associated with a slower progression of symptoms and vice-versa due to its antioxidant function. However, in the specific case of PD, Grx activity may differently impact several processes associated to disease progression (thoroughly reviewed by Gorelenkova Miller and Mieyal [161]). Some examples are given hereafter.

As previously mentioned, DJ-1 is involved in neuroprotection and is regulated via PSSG of its regulatory cysteines [156]. Grx1, via its deglutathionylase activity, regulates DJ-1 activity, avoiding its degradation which correlates with neuroprotection [162]. Indeed, Grx1 knockout mice show increased glutathionylation and loss of function of DJ-1 which, in SH-SY5Y and *C. elegans* causes dopaminergic neuron loss, a feature associated to PD [162]. In fact, in *C. elegans* deletion of the Grx1 homolog augmented loss of dopaminergic neurons and acquisition of PD-like phenotype [163].

Most interestingly, Grx1 appears to be implicated in sexual differences regarding sensitivity to parkinsonian compounds. Female mice resistance to MPTP has been linked to higher baseline levels of Grx1 compared to male counterparts in a neurodegeneration model [164]. When females were treated with an estrogen receptor antagonist, Grx1 levels in brain tissue were down regulated and susceptibility to MPTP increased [164]. The protective mechanisms is likely related to the active role of both Grx1 and Grx2 in keeping brain mitochondrial Complex I – a MPTP target – functional by reducing PSSG mixed disulphides [140,145].

Contrary to observation in neurons, overexpression of Grx1 in microglia is associated with increased neuroinflammation and PD progression. As shown by Gorelenkova Miller et al. (2016) [165], up-regulation of Grx1 in microglia cells results in proinflammatory signalling which ultimately leads to neuronal death in co-culture systems. Most interestingly, the authors also analysed the brains of PD patients for the copy number of the Grx1 gene (*GLRX*) and showed a positive association with early on-set PD. The authors suggest that the neuroinflammatory effect in microglia may overwhelm any protective effect conferred by a higher expression of Grx in neurons [165].

In the case of Alzheimer’s disease, AD, analysis of post-mortem brain samples from patients found that Grx1 levels were increased relatively to control patients whereas Trx1 levels appeared downregulated. Subsequent experiments with SH-SY5Y cells showed that amyloid beta peptide, Aß, causes a strong oxidation of both enzymes leading to Daxx export from the nucleus and cell death. On the contrary, an overexpression of either Grx1 or Trx1 offered protection against Aß toxicity [166]. Interestingly, Grx1 and Trx1 are released into the cerebrospinal fluid in AD patients especially in the early stages of the disease and are related to other AD markers such as tau [167]. So, Grx1 in the cerebrospinal fluid could be a useful biomarker for early detection of AD.

Moreover, Grx2 appeared diminished in the axonal process of hippocampal sections, of AD patients relatively to healthy controls [167]. This could have several implications particularly in the control of protein glutathionylation. In fact, analysis of AD patients showed that GAPDH, alpha enolase and tau are heavily glutathionylated comparatively to controls, signifying that hampered Grx activity could be a relevant player in AD progression [168].

In murine motor neurons and SH-SY5Y cells, overexpression of Grx1 was capable of preventing aggregation of mutant SOD1 (mutSOD1), a protein implicated in familial amyotrophic lateral sclerosis (ALS) [169]. However, this could not prevent mitochondrial damage. On the other hand, overexpression of Grx2 is capable of preventing cell death induced by mutSOD1. The action of Grx1 and 2 over mutSOD1 aggregates is believed to result from the ability to reduce disulphides between cysteine residues [170,171] but only Grx2 appears to prevent oxidative damage at the mitochondria [101]. Further research, specifically analysing patient samples, would give further elucidation on the role of Grx isoforms in ALS.

#### 4.1.4 Interaction with the thioredoxin system

The last decade saw an increasing body of evidence showing that Grxs along with GSH can function as backups for Trx reductase, securing the reduction of Trx and its downstream functions [172,173].

Indeed, Grx1 and GSH receiving electrons from NADPH via glutathione reductase, could serve as alternative reducing agents of the disulphide in Trx1 active site (Cys32 and Cys35). Also, Grx1/GSH are able to reduce a second disulphide in Trx1 between Cys62 and Cys69 [174]. Likewise, mitochondrial Grx2 is capable of reducing Trx2 but also Trx1 [173]. The catalytical efficiency of this backup mechanism, although lower than that of Trx reductase, is still enough to secure Trx1/2 reduction at physiologically relevant levels [172,173].

The significance to CNS function of such interaction is not yet know but it appears to provide the CNS with increased resistance to toxic insults, raising the threshold of toxicity of neurotoxic compounds. In fact, subsequent work in SH-SY5Y cells showed this mechanism to be involved in the development of the neurotoxicity of mercury (Hg) compounds. It was observed that in spite Hg compounds target Trx reductase at low concentrations (1 μM) due to the low pKa selenol in its active site [175,176], Trx functionality was kept until Hg levels were above 5 μM. This was attributed to the conjugated action of Grx and GSH. Indeed, experiments with primary cerebellar neurons of mice lacking mitochondrial Grx2 showed that Trx2 oxidation was much faster than in neurons from wild-type mice following exposure to ethyl-mercury. Oxidation of Trx resulting from the breakdown of this backup mechanism involving Grx resulted in ASK-1 mediated cell death [177].

### 4.2 Glutaredoxin role in cancer biology

#### 4.2.1 Regulation of the cellular cycle

The maintenance of a balanced redox environment is important for cell proliferation [178,179]. The glutaredoxin and thioredoxin systems act in this process by activating transcription factors [180,181] and reducing ribonucleotide reductase for DNA synthesis [[182], [183], [184]].

The thioredoxin system was considered the only natural hydrogen donor system for ribonucleotide reductase. However, in the thioredoxin-deficient *E. coli* mutant strain, the Grx system was found to be a second hydrogen transport system [1]. The association of both systems with the donation of reducing equivalents to RNR has led many groups to demonstrate Grx and Trx systems’ participation in tumour transformation. The first findings on the involvement of Grx came from the use of drugs that were able to inhibit the synthesis of DNA and proteins such as sesquiterpene lactones [185]-and to deplete glutathione such as l-buthionine-(S/R)-sulfoximine (BSO) [186,187]. These and other studies reporting that S-glutathionylation can modify proteins involved in cancer signalling mechanisms served as the basis for associative investigations between Grx and tumours.

In a mechanistic study with different types of tumour cell lines, the silencing of Grx1 activated the p53 signalling pathway, causing the cell cycle to stop in G1 causing senescence [188], which supported the idea that antioxidant systems, including Grx1, get upregulated in tumour cells as survival mechanism to counteract the high amount of ROS due to the changes in metabolism. This concept was also corroborated in another set of experiments with oral squamous carcinoma cells (CAL27). After exposure of these cells to Interleukin-1 beta (IL-1β, a pleiotropic cancer-inflammation-linked cytokine), an increase in Grx1 was reported, causing a decrease in the levels of ROS and conferring to oral squamous cell carcinoma, invasiveness and migration [189]. Interestingly, a mouse model with endothelial cell-specific overexpression of Grx1, after subcutaneous implantation of melanoma cells, showed increased tumour growth but inhibition of angiogenesis [190]. In another tumour model, STAT5 induced by Bcr-Abl in chronic myeloid leukemia (CML) cells inhibited the expression of Grx1, increasing ROS levels with consequently decrease of cell viability [191].

Due to the high ROS levels produced in the mitochondria [192,193], antioxidants like Grx2 are over expressed to protect mitochondrial proteins such as Complex I via glutathionylation/deglutathionylation, avoiding blocking apoptotic stimuli such as the release of cytochrome *c* to the cytosol [194,195]. However, an inverse correlation has been reported in lung cancer; after analyzing 42 cases of non-small cell lung cancer patients, it was noted that the decrease in the expression of Grx1 and Grx2 was associated with an increase in cell proliferation [196].

Over the last few decades also the expression of Grx3 has been explored in tumours. The increase in Grx3 expression was associated with several solid tumours, including an increase in proliferation in colon and lung cancer [197], migration and invasion in oral squamous cell carcinoma [198], and growth and metastasis in nasopharyngeal carcinoma [199]. In line with the studies that associated Grx1 with the cell cycle regulation, Grx3 has recently been reported as an important marker of DNA damage [200]. Grx3 acts as an upstream effector on the ATR signalling pathway, a stress-induced DNA-damage response system. In another study Pandya and co-authors reported a negative correlation between Grx3 expression and cell cycle arrest via H3 tri-methylation on lysine 27 (H3K27me3) [110,201]. This epigenetic mark is commonly associated with heterochromatin formation and, thus, with repression of gene expression. The key finding was that Grx3 knockdown led to the reduction of H3K27me3 at the *CCDN2* gene promoter. In a recent report about the vIRF1-induced oncogenesis, the authors noted an upregulation of a circular RNA (circARFGEF1), a molecule that binds and degrades miR-125a-3p [202]. Further analysis by mass spectrometry showed that the miR-125a-3p was responsible for the downregulation of Grx3 expression. In this case, the decrease in expression led to a decreased cell motility, proliferation, and angiogenesis.

#### 4.2.2 Grx and breast cancer

Studies conducted with breast tumour strains resistant to doxorubicin treatment (MCF-7 ADR(R)) have reported that resistance was associated with increased expression of antioxidant genes, including Grx [203,204]. The protective capacity against doxorubicin conferred by Grx was not associated with its redox activity, but probably connected with its ability along with thioredoxin to increase the expression of genes that metabolize xenobiotics or MDR-type receptors [205,206].

Grx3 is associated with the normal development of the mammary gland during pregnancy and lactation in mice [207] therefore, it was one of the protein of interest in the breast tumour studies. Indeed, Grx3 is overexpressed in breast cancer tissue [208] and in breast adenocarcinoma malignant phenotypes in human samples [209].

The cytotoxicity of radiotherapy is associated with the generation of reactive oxygen species and free radicals [210,211], which laid the basis for the antioxidant systems down regulation treatment studies. Despite its importance for tumour development, Grxs have not been shown to be a good marker for the response to radiotherapy in early-stage invasive breast cancer patients, like other components of redox systems [212].

#### 4.2.3 Grx and lung cancer

Glutaredoxin is widely expressed in human lung tissue, including in samples from healthy lung, parenchymal sarcoidosis, extrinsic allergic alveolitis and usual interstitial pneumonia but also in cell lines derived from the lung [213]. The most abundant isoforms at the level of mRNA are Grx1 and Grx2. However, in an investigation of the expression pattern of members of the thioredoxin superfamily in paraffinized samples from 42 patients with lung cancer, only Grx1 was marked by immunohistochemistry in histological sections [196]. In this study, all investigated proteins, with the exception of thioredoxin reductase1, correlated with the degree of differentiation of adenocarcinoma. In another study, the RNA expression of Grx3 was the most abundantly redox enzyme found in colon and lung cancer patient tissue [197]. In this study, the expression of Grx3 showed a positive correlation with that of survivin and cell survival rate. Survivin is a protein associated with the tumourigenic process due to its function as an inhibitor of apoptosis that regulates cell proliferation promoting cell survival [214,215].

A factor involved in breast tumour resistance to therapy over expression of the P-glycoprotein transporter, also appears to be present in human lung adenocarcinoma cell lines [216]. Although resistance to chemotherapy is multifactorial, a cluster of genes always expressed in cells that are resistant to treatments include zinc finger proteins, Glutaredoxin, and heat shock protein, suggesting the importance of finding a good strategy to develop Grx inhibitors.

#### 4.2.4 Grx as tumour biomarker

Different studies have examined the expression of Grx related to cell survival against ROS and as possible biomarker of tumourigenesis. For example, liver tumours have a change in the expression of redoxins depending on the progression stage [[217], [218], [219]]. In the study by Mollbrink and colleagues, with immunohistological analysis of liver paraffinized patient samples, it was observed that there was an increase in the expression of Trx1, Trx2, and Grx5 in tissue of patients with hepatocellular carcinoma (HCC) and colorectal carcinoma (CRC) in liver metastasis compared to the healthy surrounding tissue from the same patients. The Grx1 and Grx3 isoforms were observed only in CRC metastases. It is worth mentioning that among the analysed redoxins, Grx2 was elevated in patients affected by the metabolic syndrome. An expansion of this study with a larger patient sample would help to understand if the different Grxs isoforms could characterize specific cancer cell subpopulations. Another indication of the possible usage of Grx as a biomarker in specific tumours come from studies reporting Increased expression of Grx3 specifically in colon [220] and in bladder tumours [221] while Grx1 has been reported as a possible malignant marker in pancreatic duct carcinomas [222] and in the study by Zhao and collaborators, who sought non-invasive urinary markers for HCC, Grx1 expression decreased [219]. Interestingly, in a clinical phase II study in patients with advanced malignant melanoma, it was noted that the response to monotherapy with nitrosourea fotemustine could have two results that the authors correlated with the expression of different systems of glutathione reductase/glutaredoxin; i) sensibility to the drug, including full recovery, or ii) no response with no change in tumour size [223]. Even if the available data are promising, in all these mentioned studies, the sample number was too low to confirm the usage of Grx in the prognosis of different types of tumour. More comprehensive studies, including other biological samples (for example, plasma or serum), could aid in understanding if Grxs can be utilized in biomarkers for tumour stage and malignancy.

#### 4.2.5 Glutaredoxin as a chemotherapeutic target

Considering the role of Grx1 in supporting RNR activity and the reports showing upregulation of the Grx1 in specific tumours, different groups explored the possibility of targeting Grx1 as a cancer therapy strategy [224]. However, very few studies led to positive results. The more successful approaches targeting the inhibition of Grx1 by the reaction with sulfhydryl groups were represented by the fungic toxin Sporidesmin [225], the irreversible inhibitor CWR-J02 [226], and by the small molecule APR-246 (PRIMA-1Met) [184]. In the first case, the reversible inhibition by Sporidesmin was successful only in *in vitro* conditions with recombinant enzymes but did not present satisfactory activity in cellular models, due to the competition with glutathione under physiological conditions. Gorelenkova Millerand co-authorsdid a screening of more than 500 compounds and identified the molecule CWR-J02 as being active in *in vitro* conditions but, also in this case, in a cellular context, it was not selective for Grx1. APR-246 is under different clinical trials because it induces tumour cell death through reactivations of mutant p53, but it has also been shown to inhibit other cellular thiol-dependent redox systems and is therefore not selective to Grx1.

To the best of our knowledge, there are no papers in the literature describing inhibitors for any of Grx2 isoforms. A non-specific Grx3 inhibitor has recently been described, Frenolicin B [227]. It covalently modifies the sulfhydryl groups of Prx1 and Grx3, increasing the amount of reactive oxygen species and lowering the GSH/GSSG ratio in cells.

An efficient inhibitor of Grx will most likely have to display a non-reversible binding mode. The highly reactive nature of the active site cysteine, in a relatively exposed surface position, represents the major challenge because a reversible effective inhibitor should out-compete the high cellular concentration of glutathione. Therefore, a different approach to counteract this difficulty must be followed to target efficiently different Grx isoforms with anticancer drugs.

## 5 Conclusions

Although the importance of Grxs was demonstrated in several aspects of life, we are still only scratching on the surface of Grxs impact on cellular functions and the compensatory mechanism of interaction with the other redox systems. Research effort should focus in better understanding the real physiological importance of glutaredoxins, in particular of his role as a glutathionylase/deglutathionylase enzyme. In fact, protein glutathionylation has emerged as an important regulator of different protein functions but the mechanism under which the dithiol Grxs are activated and are able to recognise the protein targets is still not understood. Moreover, most of the glutathionylated proteins studied are cytosolic and not much is known about what happens in other cellular compartments. Regarding the role of monothiol Grxs the field is still developing but it has really high potential due to the indications that monothiol Grxs are key players in the iron trafficking and homeostasis.Moreover, given the already discovered function of Grx in several pathological processes its role as a drug target is certainly a hot topic but at the same time overly complex due to the biochemical characteristics of the Grxs and the unknown mechanism of activation. Finally, we would like to mention that the majority of the papers cited in this review were done in Professor Holmgren lab or by researchers that, for long or short period, visited his lab and remained fascinated by this small redoxin. Many new characteristics of Grxs have been revealed from the first discovery in 1976 but the journey to really understand the involvement of the different Grxs in signalling and diseases is still long (and exciting) and many more questions have to be answered.

## Declaration of competing interest

The authors report no conflict of interest.

## Figures and Tables

**figs1:**
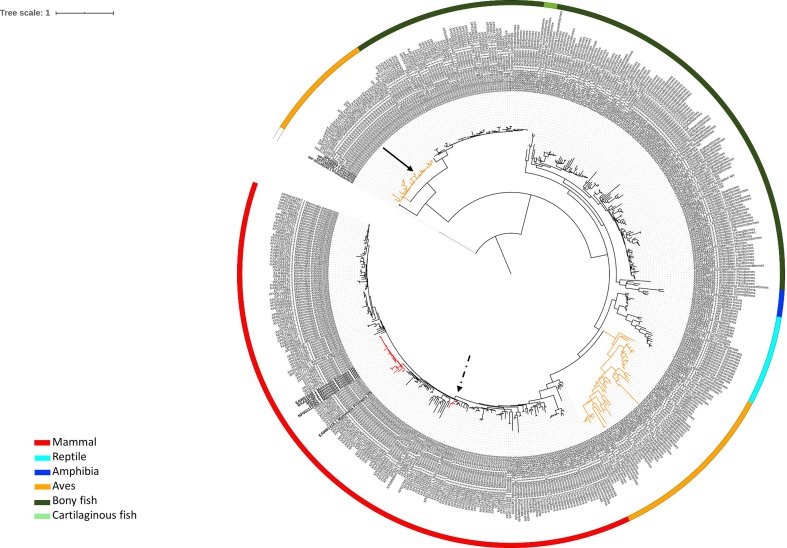


**figs2:**
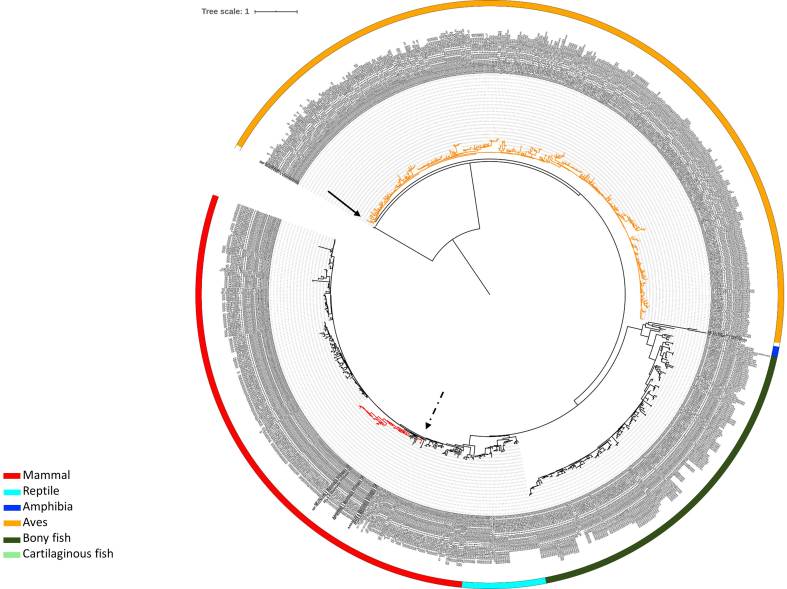


**figs3:**
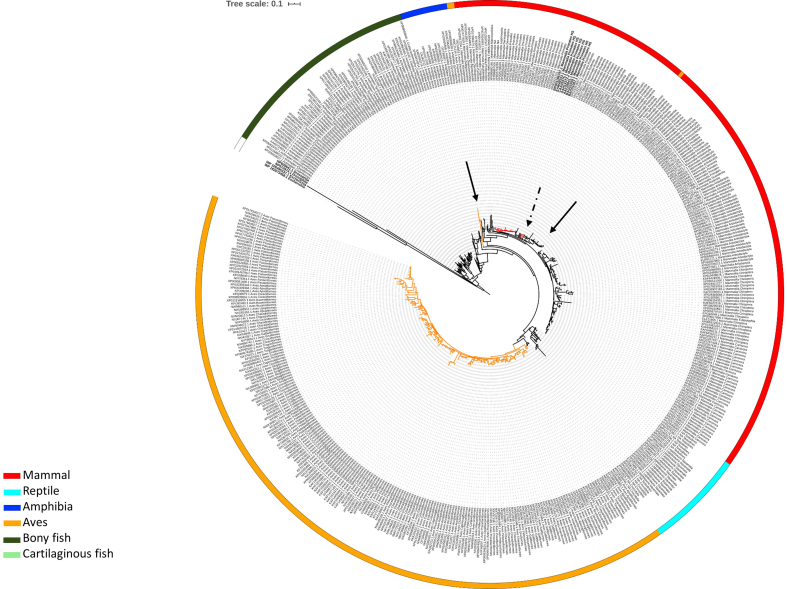


**figs4:**
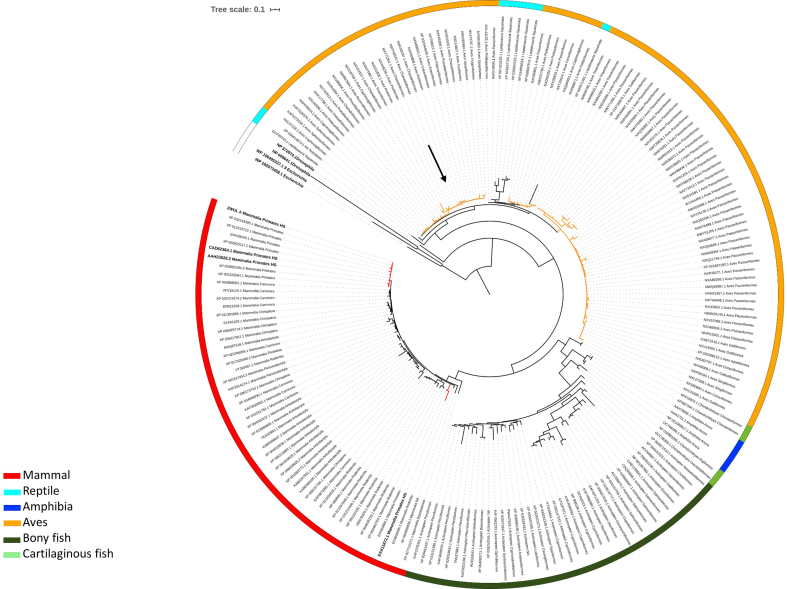


**figs5:**